# Current Status and research hotspots in the field of full endoscopic spine surgery: A bibliometric analysis

**DOI:** 10.3389/fsurg.2022.989513

**Published:** 2022-09-02

**Authors:** Guang-Xun Lin, Ming-Tao Zhu, Vit Kotheeranurak, Pengfei Lyu, Chien-Min Chen, Bao-Shan Hu

**Affiliations:** ^1^ Department of Orthopedics, the First Affiliated Hospital of Xiamen University, School of Medicine, Xiamen University, Xiamen, China; ^2^ The Third Clinical Medical College, Fujian Medical University, Fuzhou, China; ^3^ Department of Neurosurgery, the First Affiliated Hospital of Xiamen University, School of Medicine, Xiamen University, Xiamen, China; ^4^ Department of Orthopaedics, Faculty of Medicine, Chulalongkorn University, and King Chulalongkorn Memorial Hospital, Bangkok, Thailand; ^5^ Center of Excellence in Biomechanics and Innovative Spine Surgery, Chulalongkorn University, Bangkok, Thailand; ^6^ Department of Breast Surgery, The First Affiliated Hospital of Hainan Medical University, Haikou, China; ^7^ Division of Neurosurgery, Department of Surgery, Changhua Christian Hospital, Changhua, Taiwan; ^8^ Department of Leisure Industry Management, National Chin-Yi University of Technology, Taichung, Taiwan; ^9^ School of Medicine, Kaohsiung Medical University, Kaohsiung, Taiwan

**Keywords:** bibliometric, citespace, full endoscopic spine surgery, research trends, visualization

## Abstract

**Purpose:**

We aimed to comprehensively analyze the current status, hotspots, and trends in full endoscopic spine surgery (FESS) research using bibliometric analysis and knowledge domain mapping.

**Methods:**

The Web of Science database was used to screen FESS-related articles published between January 1, 1993 and June 10, 2022. The evaluation involved the following criteria: total number of articles; H-index; and contributions from countries/regions, institutions, journals, and authors.

**Results:**

A total of 1,064 articles were included. Since 2016, there have been a significant number of publications in the field of FESS. The country/region contributing the largest number of articles was China (37.8%), followed by South Korea (24%), the United States (16.1%), Japan (5.7%), and Germany (5.1%). South Korea (35) had the highest H-index, followed by the United States (27), China (22), Japan (21), and Germany (20). World Neurosurgery (15.7%) published the largest number of FESS-related articles. However, among the top 10 most cited articles, six were published in *Spine*. The author who contributed the most was S.H. Lee (5.4%), and the largest number of contributions in this field originated from Wooridul Spine Hospital (South Korea; 6.1%). Notably, six of the 10 most published authors in this field were from South Korea. Of the top five productive institutions, three were from South Korea. The keywords with the strongest citation bursts in the field of FESS were “lumbar spine,” “discectomy,” “interlaminar,” “surgical technique,” “follow-up,” “excision,” “thoracic spine,” and “endoscopic surgery.” The 10 clusters generated in this study were: “endoscopic discectomy” (#0), “thoracic myelopathy” (#1), “recurrent lumbar disc herniation” (#2), “low back pain” (#3), “cervical vertebrae” (#4), “lumbar spinal stenosis” (#5), “transforaminal lumbar interbody fusion” (#6), “radiation exposure” (#7), “management” (#8), and “lumbar spine” (#9).

**Conclusion:**

Global research on FESS is mostly concentrated in a few countries/regions and authors. South Korea has made the largest contribution to the field of FESS. Based on the most cited keyword bursts and clusters, the focus of FESS research was found to include its indications, management, and applications.

## Introduction

In recent years, percutaneous full endoscopic spine surgery (FESS) has gradually been adopted by spine surgeons owing to the following advantages: minimal invasiveness, highly effective features, increasing amount of attention from patients, and gradual expansion of its indications (1–4). The reason for the rapid development of this technology is that, compared with traditional open spine surgery, FESS does not involve massive destruction of muscle tissue, there is no need for the destruction of synovial joints and vertebral plates, and it lessens the distraction of nerve roots and dural sacs which ensures maximum stability of the spinal segment and reduces the occurrence of long-term pain and discomfort due to spinal instability and other complications (5–7). After decades of development, the use of FESS has gradually expanded from simple lumbar disc herniation (DH) to lumbar spinal stenosis and instability treatment; from lumbar to cervical and thoracic spine treatment; from pure decompression to endoscopic-assisted fusion techniques; and from the treatment of degenerative spine diseases to that of spinal trauma, infection, deformity, and tumors (8–11). With the widespread popularity of FESS, the amount of research in this field is increasing.

Bibliometric studies are commonly used to quantitatively evaluate published research and to forecast future trends in scientific research. These studies combine mathematical and statistical methods and usually aim to identify research field components, which may include authors, institutions, countries/regions, and journals. The goal of these studies is to reveal a bibliometric structure that illustrates the network between research components and contributes to the knowledge structure that is built on topic clusters related to the research field (12). By obtaining vast amounts of data in the form of knowledge maps, researchers may gain valuable insight into the trajectory of discipline growth and frontier tendencies in the field of interest. Researchers may use this method to dive deeper into research patterns and to better identify research hotspots. The findings may also be used in future research and decision-making.

Bibliometrics has been applied widely in the analysis of scientific research in various fields (13–15). Since the authors published their first bibliometric study (16) on FESS (data collected through July 2018), many FESS studies have been updated worldwide. In particular, with the recent development of biportal endoscopic spine surgery and full endoscopic spinal fusion surgery, the indications for the application of FESS have become broader, and many studies have been published on these techniques. Therefore, in this study, we aimed to perform a comprehensive assessment of the scientific research in the field of FESS worldwide through an up-to-date quantitative and qualitative analysis of the existing literature.

## Materials and methods

### Sources of data

All data were obtained from the Web of Science (WoS) Core Collection database. We searched the WoS database for articles published between January 1, 1993, and June 10, 2022. The following keywords were used to search the database: “percutaneous endoscopic spine surgery,” “percutaneous endoscopic spinal surgery,” “endoscopic cervical discectomy,” “endoscopic cervical foraminotomy,” “endoscopic cervical decompression,” “endoscopic cervical interbody fusion,” “endoscopic thoracic discectomy,” “endoscopic thoracic decompression,” “endoscopic lumbar discectomy,” “endoscopic lumbar laminotomy,” “endoscopic lumbar foraminotomy,” “endoscopic lumbar decompression,” and “endoscopic lumbar interbody fusion.” The terms “microendoscopic spine surgery,” “laparoscopic,” “thoracoscopic,” and “endonasal” were excluded.

### Data analysis

Two independent observers assessed the articles extensively based on their titles and abstracts. Disagreements were discussed and assessed by a third party. All the articles were collected and exported as plain-text files for recordkeeping and examining the cited references. The title, authors, abstract, funding, keywords, references, and other pertinent analytical information were included in each bibliographic record.

The quantity of research production was determined by the number of published articles, whereas the quality of research output was determined by the H-index and citations.

CiteSpace (Chaomei Chen, Drexel University, USA), was used to perform the bibliometric research on the data in this study (17). We used CiteSpace to identify the top authors, institutions, and countries/regions, as well as the research cooperation linkages that existed between these categories. A co-citation network analysis of authors, institutions, countries/regions, and references was performed to further investigate the research cooperation linkages. A co-word network analysis of keywords was undertaken to acquire cutting-edge information and examine trends. The frequency of the occurrence of a keyword or reference across time was denoted by co-citation relationships.

The size of nodes in a visual network diagram represents the degree of co-occurrence or citation frequency. The node connection represents the relationship between co-occurrence and co-citation. The thickness of the linkages and length between nodes reflect how closely countries/regions, institutions, and writers collaborate. The lines represent the connections between the nodes and their colors represent the year of publication.

Our research was essentially descriptive. Without statistical analysis, the quantity and ratio (percentage) of each indicator show the distribution and evolving trends in terms of different years, countries/regions, institutions, journals, and authors.

## Results

### Publication outputs

From January 1, 1993, to June 10, 2022, 1,549 articles were screened, and after a detailed review by two authors, 1,064 articles were finally identified as meeting the inclusion criteria. Among these, 940 were original articles, and 124 were review articles.

**Figure 1:**
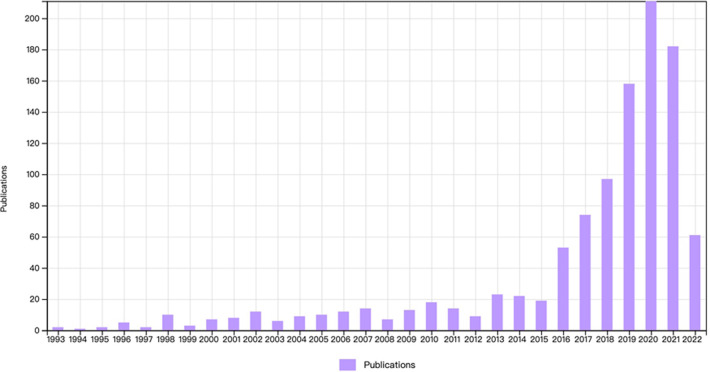
The annual trends of publications and citations.

More than 99.5% (1,059/1,064) of the articles were published in English, followed by in German (two articles), Czech (one article), French (one article), and Portuguese (one article). From 1993 to 2015, there was a period of modest development in terms of the number of publications. Following a surge in 2016, the number of publications increased significantly, reaching 211 in 2020, which is more than 100 times the number in 1993 (Figure 1). Additionally, the 1,064 articles were cited 13,404 times.

### Analysis of countries/regions

**Figure 2:**
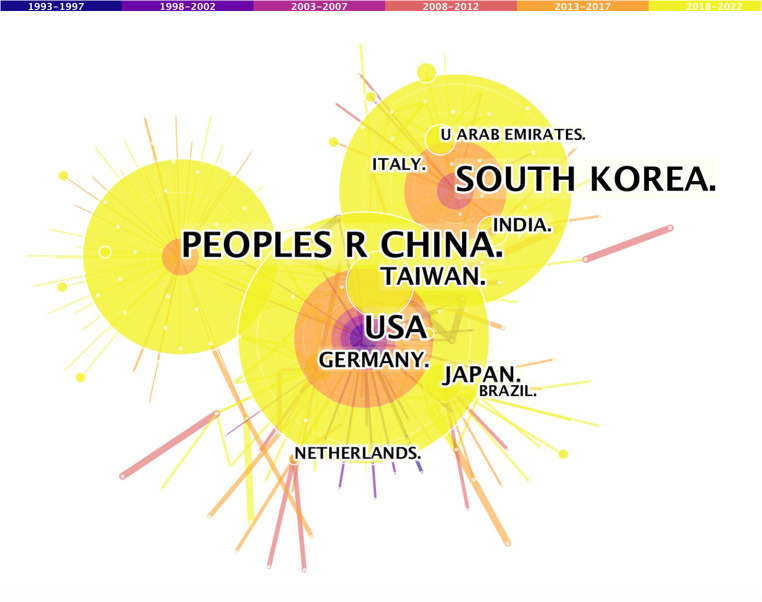
Co-operation of productive countries/regions.

The research articles on FESS were published across 49 countries/regions (Table 1). China had the highest number of publications (37.8%, 402/1,064), followed by South Korea (24%, 256/1,064), the United States (16.1%, 171/1,064), Japan (5.7%, 61/1,064), and Germany (5.1%, 54/1,064). Together, these top five countries published 88.7% of all FESS-related articles. To identify relevant signals, a co-occurrence map (Figure 2) was drawn to help researchers in detecting the cooperation linkages. There was a paucity of international collaborations among key nations in the field of FESS. Table 1 also shows the H-indices in the top five countries. South Korea had the highest H-index (35), followed by the United States (27), China (22), Japan (21), and Germany (20).

**Table 1:** Top 5 countries that contributed to research publications in the FESS field.

Rank	Country	Number	%	H-index
1	China	402	37.8	22
2	South Korea	256	24.0	35
3	USA	171	16.1	27
4	Japan	61	5.7	21
5	Germany	54	5.1	20

### Analysis of institutions

**Figure 3:**
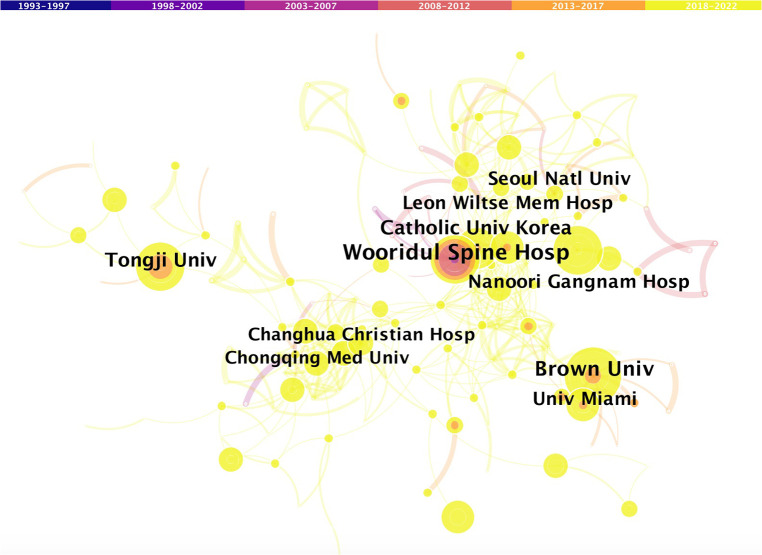
Co-operation network of productive institutions.

Table 2 ranks the institutions in terms of the number of published FESS-related articles. Wooridul Spine Hospital had the largest number of published articles (65 publications, 6.1%), followed by Brown University (57 publications, 5.4%), Catholic University of Korea (44 publications, 4.1%), Nanoori Hospital (43 publications, 4.0%), and TongJi University (41 publications, 3.9%). Among the top five productive institutions, three are in South Korea, one in China, and one in the United States. Figure 3 depicts the extent to which the institutions collaborate on FESS.

**Table 2:** Top 5 productive institutions in the FESS field.

Rank	Institution (Country)	Number	%
1	Wooridul Spine Hospital (South Korea)	65	6.1
2	Brown University (USA)	57	5.4
3	Catholic University of Korea (South Korea)	44	4.1
4	Nanoori Hospital (South Korea)	43	4.0
5	TongJi University (China)	41	3.9

### Analysis of journals

Table 3 lists the top 10 journals based on the number of articles published in the field of FESS. Of the 1,064 FESS-related articles, most were published in *World Neurosurgery* (167 articles, 15.7%), followed by *Pain Physician* (67 articles, 6.3%), *Medicine* (45 articles, 4.2%), *Neurospine* (38 articles, 3.6%), *Spine* (36 articles, 3.4%), *European Spine Journal* (28 articles, 2.6%), *BMC Musculoskeletal Disorders* (28 articles, 2.6%), *Journal of Neurosurgery: Spine* (27 articles, 2.5%), *Biomed Research International* (25 articles, 2.4%), and *Acta Neurochirurgica* (23 articles, 2.3%). It was found that nearly half (45.6%) of the FESS-related articles were published in the top 10 most prolific journals. It is reasonable to presume that these journals are the mainstays of publication in the field of FESS and that they are more open to accepting FESS-related articles.

**Table 3:** Top 10 journals in the FESS field.

Rank	Journals	Number	%
1	World Neurosurgery	167	15.7
2	Pain Physician	67	6.3
3	Medicine	45	4.2
4	Neurospine	38	3.6
5	Spine	36	3.4
6	European Spine Journal	28	2.6
7	BMC Musculoskeletal Disorders	28	2.6
8	Journal of Neurosurgery: Spine	27	2.5
9	Biomed Research International	25	2.4
10	Acta Neurochirurgica	23	2.3

### Analysis of funding

The National Natural Science Foundation of China contributed the most financial support to FESS research, with 67 grants.

### Analysis of authors

**Figure 4:**
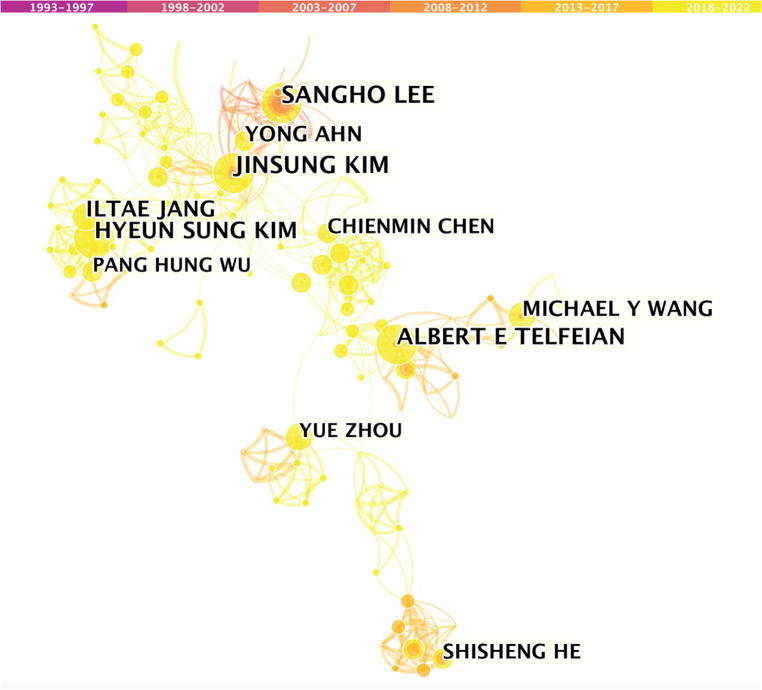
Co-operation network of productive authors.

Nearly 3,000 authors contributed to publishing the 1,064 FESS-related articles. Table 4 lists the top 10 most productive authors. S.H. Lee published the most articles (57 publications, 5.4%), followed by A.E. Telfeian (47 publications, 4.4%), J.S. Kim (42 publications, 3.9%), Y. Ahn (42 publications, 3.85%), I.T. Jang (41 publications, 3.85%), S. Ruetten (31 publications, 2.9%), H.S. Kim (30 publications, 2.9%), M.Y. Wang (29 publications, 2.7%), M. Komp (26 publications, 2.4%), and C.K. Park (26 publications, 2.4%). It is noteworthy that six of the 10 most published authors in this field were from South Korea. Figure 4 depicts the author cooperation network and further analysis showed a strong connection between these authors. It can be seen that authors who worked in the same country or who were co-authors of a study are linked in the bibliography.

**Table 4:** Top 10 productive authors in the FESS field.

Rank	Author	Number	%	Affiliation
1	S.H. Lee	57	5.4	Department of Neurosurgery, Wooridul Spine Hospital, Seoul, South Korea
2	A.E. Telfeian	47	4.4	Department of Neurosurgery, Rhode Island Hospital, The Warren Alpert Medical School of Brown University, Rhode Island, USA
3	J.S. Kim	42	3.9	Department of Neurosurgery, Seoul St. Mary's Hospital, The Catholic University of Korea, Seoul, South Korea
4	Y. Ahn	41	3.85	Department of Neurosurgery, Gil Medical Center, Gachon University, Incheon, South Korea
5	I.T. Jang	41	3.85	Department of Neurosurgery, Nanoori Hospital, Seoul, South Korea
6	S. Ruetten	31	2.9	Department of Spine Surgery and Pain Therapy, St. Anna-Hospital Herne, University of Witten/Herdecke, Herne, Germany
7	H.S. Kim	30	2.8	Department of Neurosurgery, Nanoori Hospital, Seoul, South Korea
8	M.Y. Wang	29	2.7	Department of Neurological Surgery, University of Miami Miller School of Medicine, Miami, Florida, USA
9	M. Komp	26	2.4	Department of Spine Surgery and Pain Therapy, St. Anna-Hospital Herne, University of Witten/Herdecke, Herne, Germany
10	C.K. Park	26	2.4	Department of Neurosurgery, Leon Wiltse Memorial Hospital, Suwon, South Korea

### Analysis of references

Table 5 lists the most cited publications in the field of FESS. The most cited article was by A.T. Yeung (USA), with a total of 429 citations. Five of the 10 most cited articles were from South Korea, and the remaining four were from Germany. Of the 10 most cited articles, S. Ruetten contributed to three and six were published in *Spine*.

**Table 5:** Top 10 cited articles in the FESS field.

Rank	Title	Year	Author	Journal	Citations
1	Posterolateral endoscopic excision for lumbar disc herniation - Surgical technique, outcome, and complications in 307 consecutive cases	2002	A.T. Yeung et al.	*Spine*	429
2	Transforaminal posterolateral endoscopic discectomy with or without the combination of a low-dose chymopapain: A prospective randomized study in 280 consecutive cases	2006	T. Hoogland et al.	*Spine*	193
3	Percutaneous endoscopic lumbar discectomy for recurrent disc herniation: Surgical technique, outcome, and prognostic factors of 43 consecutive cases	2004	Y. Ahn et al.	*Spine*	184
4	Use of newly developed instruments and endoscopes: full-endoscopic resection of lumbar disc herniations *via* the interlaminar and lateral transforaminal approach	2007	S. Ruetten et al.	*Journal of Neurosurgery: Spine*	181
5	A new full-endoscopic technique for the interlaminar operation of lumbar disc herniations using 6-mm endoscopes: Prospective 2-year results of 331 patients	2006	S. Ruetten et al.	*Minimally Invasive Neurosurgery*	150
6	Percutaneous endoscopic approach for highly migrated intracanal disc herniations by foraminoplastic technique using rigid working channel endoscope	2008	G. Choi et al.	*Spine*	148
7	An extreme lateral access for the surgery of lumbar disc herniations inside the spinal canal using the full-endoscopic uniportal transforaminal approach-technique and prospective results of 463 patients	2005	S. Ruetten et al.	*Spine*	146
8	Percutaneous endoscopic lumbar discectomy for migrated disc herniation: classification of disc migration and surgical approaches	2007	S. Lee et al.	*European Spine Journal*	142
9	Percutaneous endoscopic interlaminar discectomy for intracanalicular disc herniations at L5-S1 using a rigid working channel endoscope	2006	G. Choi et al.	*Neurosurgery*	134
10	Operative failure of percutaneous endoscopic lumbar discectomy: A radiologic analysis of 55 cases	2006	S.H. Lee et al.	*Spine*	133

**Figure 5:**
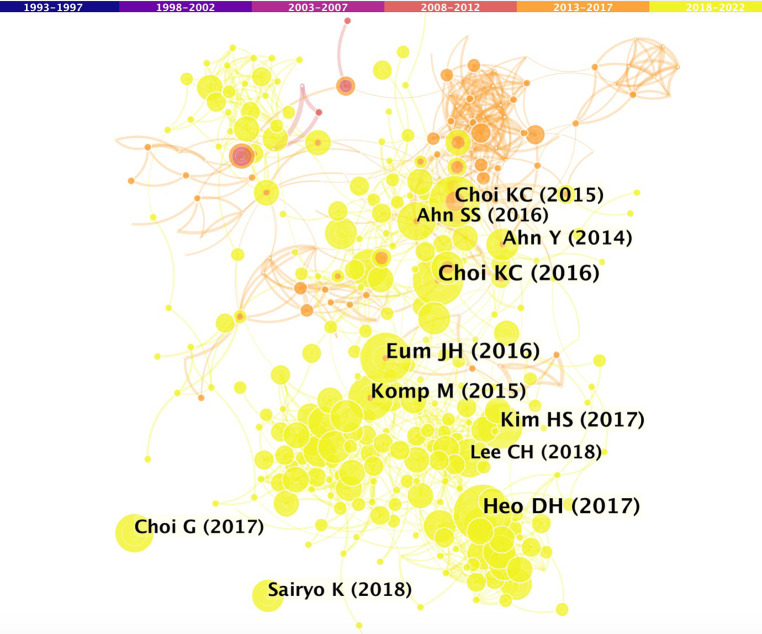
Co-operation network of cited references.

In the co-citation display analysis, the distance between references reveals the link between them in terms of co-citations. Figure 5 shows a network diagram of the cited references, which illustrates the co-citation relationships of the references. The most frequently cited article in reference lists was authored by K.C. Choi et al. (2016) (18); followed by articles authored by D.H. Heo et al. (2017) (19), J.H. Eun et al. (2016) (20), H.S. Kim et al. (2017) (21), and M. Komp et al. (2015) (22).

### Analysis of keywords and research hotspots

**Figure 6:**
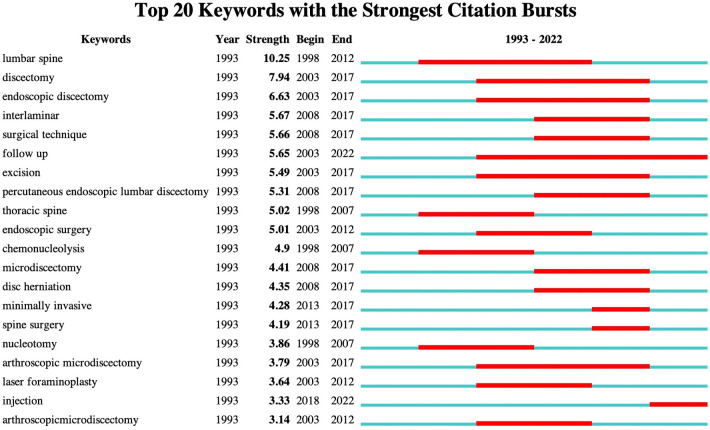
Top 20 keywords with the strongest citation bursts.

Keywords can accurately describe the topic under consideration. Summarizing high frequency and highly emerging terms in a publication can aid in describing research hotspots and trends. Figure 6 presents the top 20 keywords with the strongest citation bursts. The red bars represent the time and interval of keyword occurrence. The strongest citation burst keywords in the field of FESS were “lumbar spine,” “discectomy,” “interlaminar,” “surgical technique,” “follow-up,” “excision,” “thoracic spine,” and “endoscopic surgery.”

**Figure 7:**
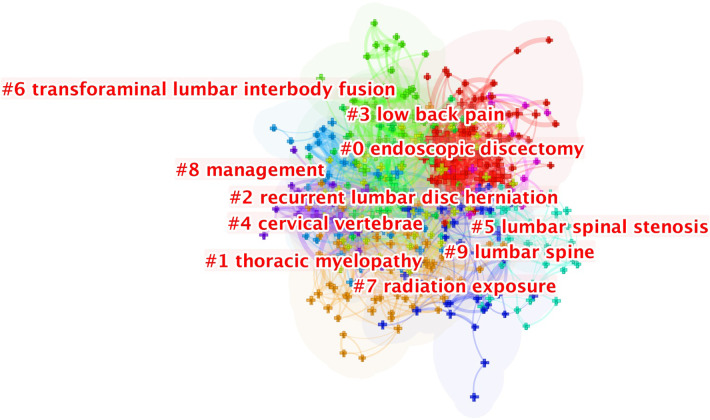
The clustering of keywords.

Keyword clustering collects words and phrases with obvious domain features and groups them into clustering objects, uses original feature extraction algorithms for text classification in order to perform domain clustering of words, and obtains generic and specific domain words by controlling the influence of word frequency. Figure 7 presents the 10 clusters generated in this study: “endoscopic discectomy” (#0), “thoracic myelopathy” (#1), “recurrent lumbar DH” (#2), “low back pain” (#3), “cervical vertebrae” (#4), “lumbar spinal stenosis” (#5), “transforaminal lumbar interbody fusion” (#6), “radiation exposure” (#7), “management” (#8), and “lumbar spine” (#9). Serial numbers were sorted by cluster size, and the field was carefully divided into several groups.

## Discussion

The current study used the WoS database and CiteSpace software to perform a bibliometric analysis of 1,064 articles on FESS published in approximately the last 30 years. The growth route from 1993 to the present was divided into two phases: 1993–2015, which was a period of gradual development, and 2016–the present, which was a period of rapid development. For decades, a great number of spine surgeons have been fascinated by the merits of FESS and have pushed for further development of this technique. Many researchers have dedicated their lives to this specific field of study and have made several significant scientific discoveries.

The surge in the number of FESS-related publications occurred in 2016. A possible reason for this is the large number of spinal endoscopic surgeons that have been trained by many spinal endoscopy-related societies around the world since 2010. Through the efforts of these groups, endoscopic spine surgery is becoming an increasingly important aspect of spine surgery and can be applied to most spinal conditions. With several additional years of practice and case accumulation, the first results began to be seen in 2016, as evidenced by a significant increase in the number of publications. Further, the development of biportal endoscopic spine surgery and full endoscopic spinal fusion procedures has greatly increased the number of spinal endoscopy publications.

Distribution analyses of countries/regions, institutions, and authors may aid in increasing collaboration and worldwide cooperation in the field of FESS. The authors discovered that the top five countries published 88.7% of all articles, implying that global FESS-related research findings were concentrated within a few countries/regions. The top five productive countries in the field of FESS were represented by the top five research institutes, three of which were in South Korea. Like in the case of other medical specialties, most of the key FESS-related research findings are uncovered by a few large countries/regions. National endoscopy-related publication outputs are provided by one or more of these national institutions. In addition, when specific authors at these institutions are examined closely, it can be found that only one or two surgeons on the team perform the bulk of the primary research. Differences in scientific output between countries/regions are multifactorial and are mainly caused by socioeconomic factors, overall research capacity, national expenditure in scientific research, and population size differences (23, 24). Furthermore, country/region level variances in specialized training in the field of endoscopic spine surgery have influenced the development of FESS techniques. Asian surgeons in China, South Korea, and Japan use spinal endoscopes more often in clinical practice and appear to perform spinal endoscopic procedures with a higher level of self-reported competence. In contrast to North America and Europe, where surgeons are still unclear about when to perform these advanced endoscopic operations, endoscopic spine surgery training appears to be more organized in Asia.

In the current study, we found that China had the largest number of publications in the field of FESS. In particular, the number of publications in China has increased dramatically over the last five years. This may be because China has an inherent demographic advantage as well as a comparable advantage in recruiting patients with spinal disorders. Moreover, China is one of the countries with the largest number of spine specialists. Their surgical and writing skills are gradually improving, thereby further increasing the output of publications (25). Furthermore, rapid economic growth has contributed to an increase in the funding allotted to the medical field and a corresponding increase in research output. Sponsorship in terms of research funding has also been significant. With 67 grants, the National Natural Science Foundation of China made the largest investment in FESS research. The number and quality of publications directly reflect the growth of the field of FESS. South Korea had the second highest number of publications after China. In addition, of the top five productive institutions, three were from South Korea. Nevertheless, among all the countries/regions contributing to the field of FESS, the H-index of published papers was the highest in South Korea. This demonstrates that the quality of research in the field of FESS is assured in the case of institutions or authors originating from South Korea. Despite being a pioneer in many biological sciences, the United States is not a leader in FESS research and had fewer publications than China or South Korea. In addition, the publication quality in the United States is also lower than that in South Korea. This may be because of the health insurance system or other economic factors. Most insurance companies in the United States do not provide adequate reimbursements for FESS. These factors may hinder the further development of FESS techniques. Additionally, the use of FESS is steadily rising in other countries/regions, such as India, Brazil, and Canada, although fewer articles may have been published because of a lack of publishing incentives. Moreover, none of the top 10 most cited articles were from China. The author of the most cited paper was A. T. Yeung from the United States. Five of the 10 most cited articles were from South Korea, and the remaining four were from Germany. This finding proves that Germany's influence in the field of FESS should not be underestimated. It can be summarized that FESS originated in Europe and the United States, while it has flourished in China, South Korea, and Japan.

It is worth noting that six of the top 10 most published authors in this discipline originate from South Korea. Further investigation revealed a clear link between these authors and they were listed as co-authors in several studies. This association has also been observed in the case of other studies. This may be characterized as a calculated and advantageous strategy.

Journal analysis may help researchers in selecting an appropriate channel for paper submission. The journal *World Neurosurgery* (15.7%), has published the largest number of FESS-related articles. In addition, of the top 10 most cited papers, six were published in *Spine*. Unfortunately, none of the top 10 FESS-related research articles with the largest number of citations were published in *World Neurosurgery*. This implies that the articles published in *Spine* may be more impactful. In addition, the FESS-related articles published in the top 10 journals accounted for 45.6% of all published FESS-related articles. These journals may be more accepting of FESS-related studies. Concurrently, articles published in these journals are more likely to be noticed and cited.

The analysis of keywords in the field of FESS revealed the focus, hotspots, and trends of research in the field. By analyzing keyword co-terminology, we identified the most prominent hotspots in the field over the past 30 years. Based on the top 20 keywords with the strongest citation bursts and top 10 keyword clusters, the research focus of FESS was found to include indications for the technique, perioperative management, and application of FESS in the treatment of various spinal diseases. After more than 30 years of development, FESS has become a common surgical approach for treating various spinal conditions; however, it must be used fairly and judiciously to maximize its advantages and avoid any associated concerns.

Many improvements have been made to FESS techniques, which has expanded its indications beyond lumbar DH to include cervical spondylosis, thoracic DH, chronic low back pain, spinal stenosis, and spinal infections.

### FESS in cervical spinal diseases

(i) Anterior approach: The primary disease that requires full endoscopic cervical surgery is cervical DH with or without foraminal stenosis. Both anterior and posterior approaches can be used for treating cervical DH. However, the surgical path is determined by the location of DH, and cervical DH in any location, including central and paracentral DH, can be treated with anterior approach cervical endoscopy (26). The advantages of cervical endoscopic surgery include a small incision; reduced risk of hematoma, infection, and vocal cord paralysis; and decreased injury to major tissues (such as the carotid artery, trachea, and esophagus) (27, 28). Therefore, this technique is useful in elderly patients or in patients with poor tolerance to anesthesia. However, the technique has some limitations. On the one hand, the percutaneous anterior approach may destroy the nucleus pulposus and may lead to postoperative narrowing or instability of the disc space; therefore, in some cases, a transcorporeal approach (the surgeon creates a safe channel from the anterior to the posterior edge of the cervical vertebrae, through which the discectomy is performed) can be used instead to achieve reduced disc destruction (29, 30). On the other hand, this technique is not suitable in cases of disc stenosis or severe calcification.
(ii) Posterior approach: The main targets of posterior endoscopic cervical foraminotomy or discectomy are herniated discs or foraminal stenosis when the primary lesion is located lateral to the spinal cord (31). The main indications for posterior approach cervical endoscopy are as follows: lateral herniated or paracentral herniated cervical DH and unilateral cervical foraminal stenosis combined with intractable cervical radiculopathy (32). According to a previous randomized trial, in cases with appropriate indications, posterior approach cervical endoscopy can be an effective alternative to traditional open surgery (33).

### FESS for thoracic spinal diseases

According to the literature, FESS resulted in favorable clinical outcomes when used to treat thoracic DH, thoracic spinal stenosis, and ossification of the yellow ligament of the thoracic spine (34, 35). Establishing good working access is a key step in percutaneous endoscopic posterolateral access thoracic discectomy, and with the help of three dimensional (3D) computed tomography navigation, bony access and precise localization of the lesion can be better established (36). The full endoscopic technique has a magnifying effect on visual field and uses radiofrequency coagulation for securing small vessels and bleeding points during surgery to ensure a clear field of view which enables precise excision of the lesion, reduces damage to the surrounding soft tissues and bony structures, and effectively prevents postoperative complications, such as postoperative adhesions and spinal instability.

### FESS for lumbar spinal diseases

(i) Transforaminal FESS is the most representative endoscopic procedure and is widely used. The basic concept underlying this technique is gaining access to the disc lesion directly through the Kambin triangle while preserving the normal anatomic tissue, which can be performed under local anesthesia and can reduce adjacent segmental lesions. The initial indication is simple lumbar DH. With the development of endoscopic techniques and instruments, their practical applications have expanded to include migrated, recurrent, and even partially calcified DH (37, 38). Furthermore, in recent years, many reports on transforaminal FESS for treating lateral recess or foraminal stenosis have been published (39, 40).
(ii) Interlaminar FESS was initially developed to treat herniated discs at L5-S1 because a transforaminal approach is difficult in patients with high iliac crests and because there is sufficient space between the laminae at the L5-S1 level to perform decompression while preserving the paravertebral muscles and most of the laminae (41). In the treatment of lumbar spinal stenosis, the transforaminal interlaminar approach is suitable in patients with lateral recess stenosis and central canal stenosis, and decompression can be performed bilaterally with a unilateral approach in patients with central canal stenosis with intermittent claudication as the main symptom (42, 43). Foraminal DH, extreme posterolateral DH, and DH with segmental instability are contraindications for interlaminar FESS (44).
(iii) In addition, the use of special approaches, such as translaminar (45), transpedicular (46), and transiliac (47) approaches, during full endoscopic techniques has been reported.

### Full endoscopic spinal fusion surgery

Endoscopic advances have been clearly demonstrated in decompression surgery, and in recent times, endoscopic fusion procedures have been frequently reported (48, 49). Full endoscopic spinal fusion surgery is a minimally invasive technique that is one of the landmarks in the advancement of spinal endoscopic technology; it has led to the development of comprehensive endoscopic spinal fusion procedures with more delicate and precise surgical techniques (50, 51). Under the same premise followed in the case of indications for lumbar fusion surgery, the recent clinical efficacy of this procedure has been satisfactory. Recently, some researchers have attempted to perform a full endoscopic anterior cervical decompression and fusion procedure (52, 53). However, this procedure still has a steep learning curve, long initial surgical time, and a high complication rate. To complete the surgery in a safer, more efficient, and minimally invasive manner, many specialists have improved and innovated the surgical techniques, accesses, and instruments.

### Biportal endoscopic spine surgery

The concept underlying unilateral biportal endoscopic spine surgery is similar to that involved in arthroscopic surgery, in which two different channels placed in the endoscopic system are used along with the working channel (54). The endoscopic channel is used to advance a 0° or 30° endoscope in order to obtain a surgical field of view, while the instrument channel is used for surgical instrument access. The surgical approach is similar to that used with microendoscopic systems; however, it involves the use of saline as a medium, flexible use of instruments, operation of most instruments with existing open surgical tools, a shorter learning curve than that associated with single-portal endoscopes, performance of most procedures under general anesthesia, use of various instruments for assistance, and free handling of instruments (55, 56). 3D endoscopy is also used to obtain depth-of-field surgical images (57). Compared with single-portal endoscopy, biportal endoscopy is slightly more disruptive to the spinal anatomy but is more efficient in decompression. Therefore, many clinicians use this technique for multilevel spinal decompression and fusion (58–60).

### Limitations

First, this bibliometric study was limited to published resources retrieved from the WoS database. Second, because bibliometric data evolve, indexing delays may have resulted in minor variations in search results. Third, regardless of merit, publications with repeated titles or titles not directly relevant to FESS may have been deleted owing to selection bias. Finally, because only papers from approximately the past 30 years were included, valuable publications from earlier years may have been omitted. Despite these limitations, our data provide information on the features of FESS-related investigations as well as on the trends in the citation of published articles.

## Conclusions

A bibliometric approach was used to analyze the quantity and quality of FESS-related publications and research hotspots. According to our study, the number of FESS-related publications has increased significantly since 2016. Most publications on FESS are limited to a few countries/regions and institutions. China has the highest number of publications, while South Korea has the highest impact as assessed by the H-index. However, the contributions of the United States, Japan, and Germany should not be overlooked. The author who contributed the most was S.H. Lee, and the largest number of contributions to this field originated from Wooridul Spine Hospital. *World Neurosurgery* published the largest number of FESS-related articles, but the articles published in *Spine* may be more impactful. Based on the most cited keyword bursts and clusters, the focus of FESS research was found to include its indications, management, and applications.

## Data Availability

The original contributions presented in the study are included in the article/Supplementary Material, further inquiries can be directed to the corresponding author/s.
